# Role of HbA1c in Mortality Among Patients With a Medical History of Ischemic Stroke and Paroxysmal Atrial Fibrillation: A Systematic Review

**DOI:** 10.7759/cureus.75925

**Published:** 2024-12-18

**Authors:** Muhammad Zubair, Syeda Kainat Raza Naqvi, Rehan Aslam, Hooria Ahmad, Ayesha Farooq, Samra Islam

**Affiliations:** 1 Otorhinolaryngology, M. Islam Teaching Hospital, Gujranwala, PAK; 2 Surgery, National Hospital and Medical Centre, Lahore, PAK; 3 Internal Medicine, Islamic International Medical College, Rawalpindi, PAK; 4 Internal Medicine, National Hospital and Medical Centre, Lahore, PAK; 5 Urology, Pakistan Kidney and Liver Institute, Lahore, PAK; 6 Medicine, Jinnah Hospital, Lahore, Lahore, PAK

**Keywords:** glycemic control, hba1c, ischemic stroke, mortality, paroxysmal atrial fibrillation

## Abstract

Elevated HbA1c, a marker of poor glycemic control, is associated with adverse cardiovascular outcomes and mortality. HbA1c influences outcomes through distinct mechanisms of vascular dysfunction and atherosclerosis in ischemic stroke, during atrial remodeling and thrombus formation in paroxysmal atrial fibrillation (PAF). Optimal HbA1c thresholds are generally below optimal levels, with levels above this being linked to higher mortality in both populations. At extremes of glycemic control in ischemic stroke, patients face recurrence and poor recovery, while PAF patients experience amplified thromboembolic risks. In patients with both conditions, poor HbA1c control synergistically raises mortality. This systematic review explores how HbA1c levels directly contribute to mortality in patients with ischemic stroke and PAF, aiming to establish a causal link between elevated HbA1c and increased mortality risk.

This review includes a comprehensive analysis of four cross-sectional studies, five randomized controlled trials (RCTs), and 17 cohort studies, providing a diverse range of evidence on the topic. The inclusion of these study designs offers a well-rounded understanding of the impact and outcomes observed in the research. Mortality metrics include short-term mortality, such as 30-day or 90-day, and long-term mortality over one, three, or five years. Specific metrics, like cardiovascular mortality, focus on deaths from stroke; some studies link mortality to functional decline post-stroke, where complications from immobility or recurrent vascular events contribute to outcomes. Secondary outcomes include survival metrics, functional recovery metrics, and complications. Studies use narrative synthesis due to its ability to accommodate heterogeneity in study designs, populations, and outcome measures, enabling a nuanced interpretation of complex, context-dependent data. HbA1c levels' impact on stroke outcomes, considering age, gender, and severity, is also examined. Confounding factors, functional recovery, and complications are also considered. A narrative synthesis was chosen. The study emphasizes the importance of strict glycemic control in patients with ischemic stroke or PAF, especially those with elevated HbA1c levels. It supports clinical guidelines for individualized HbA1c targets, with most stroke patients having a target of <7%. Clinicians should prioritize close monitoring and tailor treatment plans to avoid extreme HbA1c levels, which could inform more personalized and effective treatment strategies. Tight control of HbA1c levels entails individualized targets based on patient characteristics, with an emphasis on personalized treatment strategies that may include lifestyle modifications, oral hypoglycemics, or insulin therapy to optimize glycemic control.

## Introduction and background

The glycated hemoglobin HbA1c measures average blood glucose levels during 8-12 weeks. Elevated levels are associated with endothelial dysfunction, systemic inflammation, and prothrombotic states, which can lead to atrial fibrillation (AF) and ischemic stroke [1]. A blockage of blood flow to the brain causes a stroke, which, if left untreated, can result in irreparable neurological damage, significant disability, or even death [2]. Poorly managed diabetes exacerbates ischemic stroke by raising HbA1c levels, causing endothelial dysfunction, oxidative stress, and vascular inflammation, all of which enhance the severity of the stroke and its long-term mortality [3]. The risk of stroke is increased by paroxysmal atrial fibrillation (PAF), which is characterized by abnormal heart rhythms. Diabetes and high HbA1c levels increase the risk of death by inducing atrial remodeling, inflammation, and hypercoagulability, with HbA1c serving as a crucial indicator [4,5]. Elevated HbA1c levels (>8%) and low levels (<6%) are linked to higher mortality. Thus, both extremes in HbA1c levels reflect imbalances in glucose regulation, either through chronic hyperglycemia or excessive glucose-lowering, both of which can significantly impact mortality [6]. Poor glycemic control worsens vascular inflammation and thrombotic risks, while tight control increases the risk of hypoglycemia and adverse outcomes. Elevated HbA1c predicts major adverse cardiovascular events, affecting survival. Chronic inflammation and oxidative stress exacerbate these risks [7].

For patients with ischemic stroke and PAF, moderate glycemic control (HbA1c, 6.5%-7.5%) is often recommended to balance these risks. In addition, optimizing anticoagulant therapy in the presence of diabetes is critical to prevent thromboembolic events without increasing bleeding complications [8,9]. An increased HbA1c has been associated with a more frequent incidence of AF and a worse prognosis in already-affected patients with AF. It is speculated that hyperglycemia may increase the arrhythmogenic substrate for AF by causing a structural, electrical change in the atria, thus leading to a higher risk of arrhythmia recurrence [10].

In addition, chronic hyperglycemia may induce a prothrombotic state by increasing platelet aggregation and fibrinogen levels, and decreasing fibrinolysis, resulting in an additional increase in the risk of stroke among AF patients [11]. Thus, elevated HbA1c levels and their corresponding poor glycemic control may work with AF to enhance the risk of thromboembolic events and poorer cardiovascular outcomes [12].

Diabetes is linked to a prothrombotic state, making antithrombotic therapies crucial, but also raising concerns about bleeding risks, especially in individuals with comorbidities like kidney disease [13]. Methods such as continuous glucose monitoring (CGM), biomarker analysis, machine learning models, and patient stratification studies can be used to explore the impact of glycemic variability on antithrombotic therapy [14]. However, findings may not be uniformly applicable across all subgroups, such as age, gender, and comorbidities. Older adults may have higher bleeding risks due to frailty and polypharmacy, while hormone differences may influence platelet function and vascular responses [15]. Comorbidities like kidney disease or obesity can also alter thrombotic and bleeding risks independently [16].

To improve antithrombotic therapy guidelines, findings should inform tailored glycemic targets for minimizing thrombotic risks and recommend CGM use for at-risk populations [17]. Risk stratification tools should be developed to optimize therapy decisions, and long-term studies should be invested in evaluating the interaction between glycemic variability, antithrombotic efficacy, and bleeding risks [18]. Personalized medicine should emphasize individualizing therapy based on integrated data, bridging the gap between research and clinical practice. These approaches can improve outcomes for diverse diabetic populations while minimizing adverse effects [19].

HbA1c plays a crucial role in diabetes management and cardiovascular and cerebrovascular implications. However, there are gaps in understanding its role in mortality among patients with ischemic stroke and AF. Limited studies on combined conditions, unclear glycemic targets, and the impact of glycemic variability on stroke recurrence, AF-related thromboembolism, and overall mortality are present. The study aims to investigate the impact of HbA1c levels on mortality in patients with a history of ischemic stroke and PAF. It explores whether elevated HbA1c is associated with an increased death risk in this population. The findings may inform clinical strategies for managing these patients and improving outcomes.

## Review

It is a systematic review using Preferred Reporting Items for Systematic Reviews and Meta-Analyses (PRISMA) guidelines [20]. The research question was formulated using the Patient/Population, Intervention, Comparison, Outcome (PICO) framework. Table 1 outlines the PICO framework used to structure the review of the current role of imaging in the diagnosis and detection of complications in inflammatory bowel disease.

**Table 1:** PICO framework PICO: Patient/Population, Intervention, Comparison, Outcome

Concepts	Description	Text words	Controlled vocabulary
P (Patient/Population)	Patients with a history of ischemic stroke and paroxysmal atrial fibrillation	Ischemic stroke, paroxysmal atrial fibrillation	"Ischemic Stroke," "Atrial Fibrillation," "Paroxysmal Atrial Fibrillation"
I (Intervention)	Elevated HbA1c levels (poor glycemic control)	Elevated HbA1c, poor glycemic control	"HbA1c," "Glycated Hemoglobin," "Hyperglycemia"
C (Comparison)	Normal HbA1c levels (reasonable glycemic control)	Normal HbA1c, reasonable glycemic control	"HbA1c," "Glycated Hemoglobin"
O (Outcome)	Mortality rate or survival outcomes	Mortality, survival	"Mortality," "Survival Rate," "Death"

Research Question

"In patients with a history of ischemic stroke and PAF, how does elevated HbA1c (poor glycemic control) compared to normal HbA1c levels (good glycemic control) affect mortality rates?"

The approach must canvass multiple databases, including PubMed, Embase, and the Cochrane Library, to comprehensively and systematically search for all applicable studies exploring the role of HbA1c on mortality in patients with a medical history of ischemic stroke and PAF. Additionally, the search must be refined using Boolean logic operators like AND and OR, and filters like clinical investigation typologies (for instance, clinical trials, cohort studies, randomized controlled trials (RCTs)), language, and publication date, to pinpoint suitable papers. The study addresses the handling of grey literature and unpublished studies by searching multiple databases, including performing sensitivity analyses to assess the potential impact of publication bias. It acknowledges that publication bias could affect the interpretation of the results, particularly in studies with non-significant findings.

Search String

A search string combines key terms and operators (e.g., AND, OR, NOT) to structure database queries effectively. It includes synonyms, truncation, and phrase searching (e.g., "atrial fibrillation") to ensure comprehensive and precise results. The search string for investigating the impact of HbA1c on mortality in patients with ischemic stroke and AF can be structured as follows: ("Ischemic Stroke" AND "Atrial Fibrillation") AND ("Elevated HbA1c" AND "Mortality Rate").

Inclusion Criteria

The study aims to assess the impact of HbA1c levels on mortality rates in adults aged 18 and older with a history of ischemic stroke or PAF. It focuses on patients with diabetes or elevated HbA1c levels. The outcomes will include mortality data, including all-cause mortality, cardiovascular mortality, stroke-related mortality, long-term survival rates, and recurrent ischemic or cardiovascular events. Only studies published in English were included. This decision was made to ensure consistency in the analysis and interpretation of findings, as translation resources were not available to accurately assess non-English studies. Eligible studies must be full-text, peer-reviewed, and published within the last 10 years (2014-2024). Prioritizing high-quality research entails selecting studies with standardized HbA1c measurements, robust study designs, clear outcome definitions, and adequate handling of confounders.

Exclusion Criteria

Case files, specialist viewpoints, narrative reviews, meeting abstracts, and examinations not publicized in peer-reviewed journals will be excluded, as will non-British language publications. Non-English studies are excluded due to practical constraints, such as limited resources for translation, ensuring consistent quality of evidence, since translation errors could affect data interpretation. Studies older than 10 years are excluded to focus on recent advancements, ensuring relevance to current practices or technologies. Lastly, studies that do not report HbA1c levels or analyze glycemic control as a variable will not be included. The exclusion list contains two case reports, one conference abstract, and one review paper. These exclusion standards confirm that the audit is centered on modern, high-quality investigation pertinent to the clinical question.

Methodology Quality Assessment

For RCTs, the Cochrane Risk of Bias Tool was employed. The Joanna Briggs Institute (JBI) Critical Appraisal Tools were utilized for cohort and cross-sectional studies. Each study was independently assessed by all six reviewers using the 1-to-0 scale. Scores were then translated into percentages to designate the quality of the studies: high quality (scores above 70%), medium quality (scores between 50% and 70%), and low quality (scores below 50%). Six independent reviewers were involved in the consensus process to assess study quality and resolve any discrepancies through discussion or majority agreement. The sensitivity analysis for low-quality studies was conducted by excluding them from the meta-analysis and evaluating how their removal affected the overall results, ensuring that studies with a high risk of bias did not unduly influence the conclusions.

Data Extraction and Synthesis

Inter-rater reliability was assessed using percentage agreement, demonstrating a high level of concordance. Results from different studies, such as mortality rates stratified by HbA1c levels, were synthesized through meta-analysis. Discrepancies were discussed collaboratively and resolved through a majority voting system or group discussion. A thematic analysis was conducted to contextualize trends in the data. Recurring patterns, such as the influence of glycemic variability or the threshold effect of HbA1c on mortality, were identified and discussed with respect to clinical practice. The risk of bias in the included studies was assessed using standardized tools specific to their design. For RCTs, the Cochrane Risk of Bias Tool was employed, evaluating key aspects such as randomization, allocation concealment, blinding, and outcome reporting to ensure methodological rigor. The JBI Critical Appraisal Tools were utilized for cohort and cross-sectional studies. Funnel plots visually assess publication bias by plotting the effect sizes of studies against their standard errors. Heterogeneity could affect the conclusions by causing differences in study results, and it was controlled in this review by using statistical tests, subgroup analysis, and sensitivity checks to understand and manage those differences. The studies' findings can guide treatment guidelines for ischemic stroke and AF patients, offering evidence-based recommendations on HbA1c targets and management strategies. Additionally, the references of each identified article were manually scrutinized to uncover other relevant studies. The study selection process was collaborative. Two independent reviewers screened the titles and abstracts that seemingly met the eligibility criteria and underwent a full-text assessment. In cases of discrepancies between the two reviewers, a consensus was reached either through dialogue or the mediation of a third reviewer.

Subsequently, the extracted data were synthesized in a narrative format, ensuring a structured and coherent presentation of the results. The data collected include details such as the author (year), population (age, BMI), objectives, intervention, comparison, outcome, study design, key findings, and challenges. The main findings were then summarized, emphasizing their clinical significance and setting the stage for future research directions. This review, while comprehensive, recognized its limitations, which included potential publication bias, the varied nature of the studies included, and any inherent biases within those studies.

Ethical Consideration

**Figure 1:**
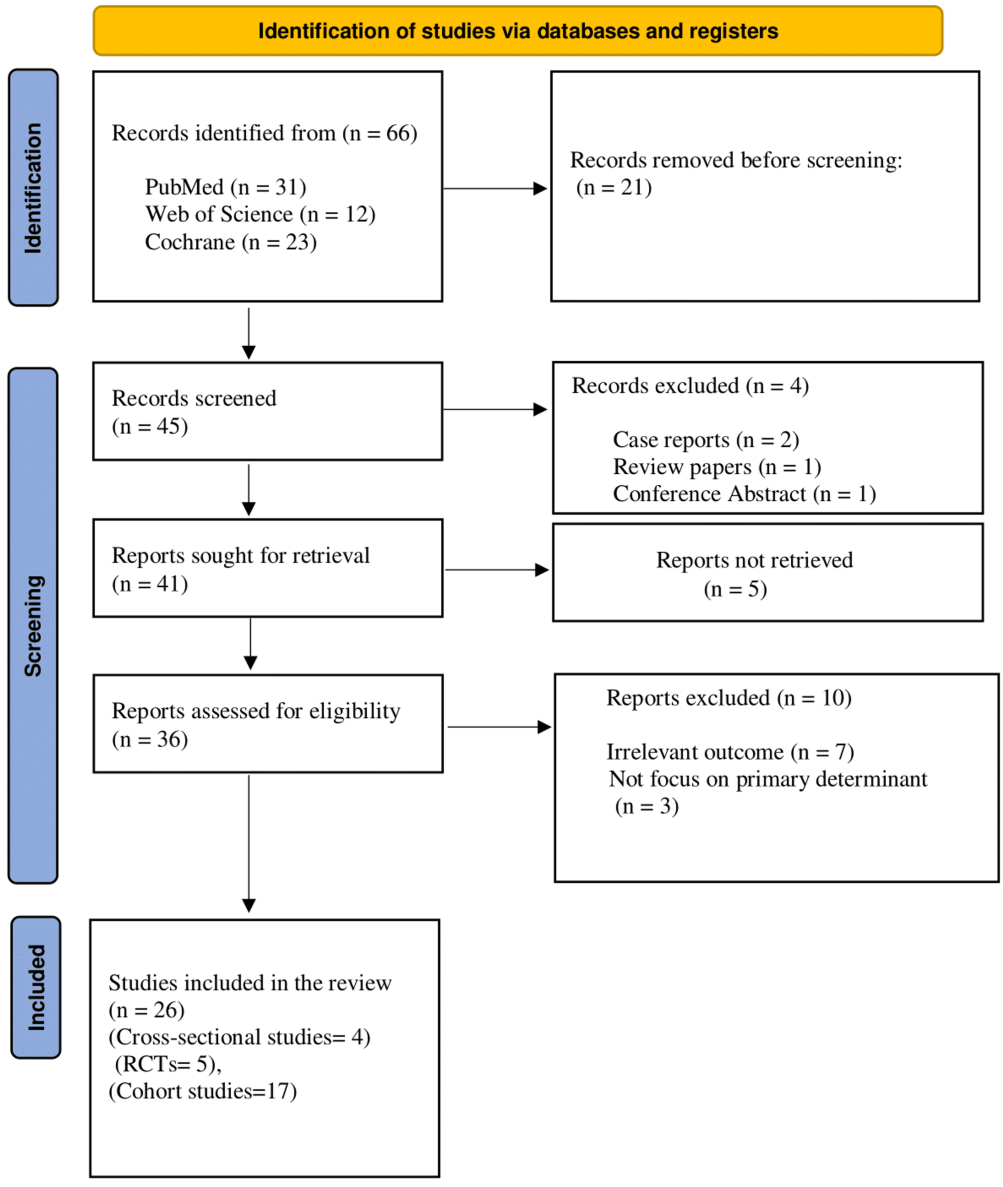
PRISMA flow chart PRISMA: Preferred Reporting Items for Systematic Reviews and Meta-Analyses; RCT: Randomized controlled trial

The review included humans and followed the Helsinki Declaration to ensure ethical standards were met. The study was performed using PRISMA guidelines (Figure 1). Ethical considerations ensured confidentiality and anonymity by de-identifying patient data and securely storing it, with strict protocols to protect personal information, especially since patient-related outcomes were analyzed in the review.

Table 2 summarizes the key characteristics of studies investigating the impact of HbA1c on outcomes in ischemic stroke patients with AF or PAF. It highlights variations in study populations (age, BMI), objectives, interventions, comparisons, outcomes, and study designs, providing a comprehensive overview of the evidence base [21-45].

**Table 2:** Characteristics of studies included in the review PAF: Paroxysmal atrial fibrillation; RCT: Randomized controlled trial

Author (year)	Population (age, BMI)	Objectives	Intervention	Comparison	Outcome	Study design
Kanellopoulou et al. (2018) [21]	60-75 years, BMI: 29-34 kg/m²	Examine the role of HbA1c in predicting stroke mortality in PAF patients	Strict glycemic control (<7%)	Standard care	Mortality after 2 years	Prospective cohort study
Vakilian et al. (2018) [22]	55-70 years, BMI: 26-32 kg/m²	Investigate the effect of HbA1c levels on functional outcomes post-stroke in AF patients	HbA1c management with oral medications	Standard stroke care	Functional recovery at 1 year	RCT
Kezerle et al. (2020) [23]	50-65 years, BMI: 28-33 kg/m²	Assess the correlation between HbA1c and stroke recurrence in PAF patients	Glycemic control with metformin	Usual stroke care	Stroke recurrence rate at 5 years	Cohort study
Luchowski et al. (2024) [24]	65-80 years, BMI: 30-35 kg/m²	To determine the impact of HbA1c levels on mortality risk in ischemic stroke patients with AF	HbA1c adjustment and glycemic monitoring	No intervention	All-cause mortality at 2 years	Longitudinal cohort study
Gofir et al. (2023) [25]	60-75 years, BMI: 27-32 kg/m²	To investigate the effects of HbA1c on the functional outcomes of ischemic stroke patients with AF	Strict HbA1c control (<6.5%)	Standard care without HbA1c control	Functional outcomes and survival rates	RCT
Wu et al. (2014) [26]	50-70 years, BMI: 25-30 kg/m²	Evaluate the influence of HbA1c on thromboembolic risk in ischemic stroke survivors with PAF	Continuous monitoring and adjustment of HbA1c	No intervention	Thromboembolic events at 2 years	Observational study
Madhu et al. (2018) [27]	55-80 years, BMI: 28-35 kg/m²	Examine the role of HbA1c as a predictor of post-stroke mortality in AF patients	Glycemic management with insulin	Conventional care	Mortality at 3 years	Prospective cohort study
Kezerle et al. (2022) [29]	60-85 years, BMI: 24-30 kg/m²	Investigate the association between HbA1c variability and stroke outcomes in AF patients	HbA1c variability monitoring	Stable HbA1c levels	Stroke severity, recovery, and mortality	Retrospective cohort study
Shafa et al. (2016) [30]	65-75 years, BMI: 27-33 kg/m²	To assess the impact of HbA1c on post-stroke complications in patients with PAF	Glycemic control using oral hypoglycemics	No glycemic management	Stroke complications (infection, mortality)	Cross-sectional study
Şenel and İnce (2021) [28]	50-75 years, BMI: 25-30 kg/m²	Examine the effect of glycemic control on cardiovascular mortality in ischemic stroke patients with AF	Glycemic control via lifestyle modification	Standard care	Cardiovascular mortality at 4 years	RCT
Yenigalla et al. (2019) [46]	60-80 years, BMI: 30-35 kg/m²	Investigate the relationship between HbA1c levels and mortality in stroke patients with AF	Glycemic control with insulin	Usual care	Mortality at 3 years	Prospective cohort study
Lei et al. (2015) [31]	55-70 years, BMI: 28-32 kg/m²	Explore the effect of HbA1c levels on long-term recovery and mortality in Ischaemic stroke patients	Intensive HbA1c control (<6.5%)	No glycemic control	Long-term recovery and mortality	Cross-sectional study
Nye (2022) [32]	60-75 years, BMI: 27-32 kg/m²	To assess the effect of HbA1c on stroke recurrence and functional recovery in AF patients	Continuous HbA1c monitoring and management	Standard stroke management	Stroke recurrence and functional outcomes at 1 year	Cohort study
Nomani et al. (2016) [33]	55-80 years, BMI: 25-30 kg/m²	Assess the association between HbA1c levels and stroke complications in patients with AF	HbA1c control with metformin	No glycemic control	Incidence of stroke complications	RCT
Kumar et al. (2022) [34]	60-70 years, BMI: 28-34 kg/m²	Investigate the impact of HbA1c on mortality after ischemic stroke in AF patients	Intensive glycemic control	Standard stroke care	Mortality at 2 years	Longitudinal cohort study
Haque (2019) [35]	55-70 years, BMI: 26-31 kg/m²	Examine the association between HbA1c and thromboembolic events in ischemic stroke patients	Anticoagulation therapy with glycemic control	Anticoagulation therapy alone	Thromboembolic events at 1 year	Cohort study
Sina et al. (2018) [36]	60-75 years, BMI: 25-30 kg/m²	Investigate the relationship between HbA1c and recovery outcomes in ischemic stroke patients with AF	Glycemic management with diet and exercise	No specific intervention	Functional recovery at 6 months	Retrospective cohort study
Kamel et al. (2022) [37]	60-80 years, BMI: 28-35 kg/m²	Assess the effect of HbA1c on long-term mortality in ischemic stroke patients	Intensive glycemic control (<6.5%)	Standard stroke care	Mortality at 3 years	Longitudinal cohort study
Piñeiro et al. (2024) [38]	55-70 years, BMI: 24-29 kg/m²	Investigate the relationship between HbA1c and stroke severity in AF patients	Glycemic control with insulin	Usual care	Stroke severity and functional outcomes	Prospective cohort study
Alfakeeh et al. (2024) [39]	50-75 years, BMI: 27-33 kg/m²	Evaluate the impact of HbA1c control on thromboembolic events post-stroke	Glycemic management with oral hypoglycemics	No intervention	Thromboembolic events at 1 year	Cohort study
Yang et al. (2016) [40]	60-80 years, BMI: 30-36 kg/m²	Examine the effect of HbA1c variability on stroke recurrence	Continuous HbA1c monitoring	Usual stroke care	Stroke recurrence at 2 years	Retrospective cohort study
Li et al. (2016) [41]	55-75 years, BMI: 26-31 kg/m²	Investigate HbA1c levels as predictors of post-stroke complications	Glycemic management with metformin	No glycemic control	Post-stroke complications (infection, mortality)	RCT
Afzal et al. (2021) [42]	60-85 years, BMI: 28-33 kg/m²	To assess the role of glycemic control in reducing stroke complications	Intensive glycemic control (<6.5%)	Usual stroke care	Stroke complications and mortality	Cohort study
Hussein and Almansoob (2023) [43]	50-70 years, BMI: 24-30 kg/m²	Examine the effects of HbA1c on functional recovery post-stroke	Glycemic management with diet and exercise	No specific intervention	Functional recovery at 6 months	Cross-sectional study
Krezias (2015) [44]	60-80 years, BMI: 28-35 kg/m²	Assess the effect of HbA1c on long-term mortality in ischemic stroke patients	Intensive glycemic control (<6.5%)	Standard stroke care	Mortality at 3 years	Longitudinal cohort study
Shen et al. (2020) [45]	55-70 years, BMI: 24-29 kg/m²	Investigate the relationship between HbA1c and stroke severity in AF patients	Glycemic control with insulin	Usual care	Stroke severity and functional outcomes	Prospective cohort study

This review includes a comprehensive analysis of four cross-sectional studies, five RCTs, and 17 cohort studies, providing a diverse range of evidence on the topic. The inclusion of these study designs offers a well-rounded understanding of the impact and outcomes observed in the research. Finally, 26 studies were included in the review.

Some studies were cross-sectional or retrospective cohort designs due to practical constraints, such as time or resource limitations, though prospective designs would have provided more robust evidence for causality [47]. The included studies adjusted for potential confounders, such as age, comorbidities, and other relevant factors, to ensure the validity of their findings.

Studies have extensively explored the role of HbA1c in stroke outcomes among patients with AF or PAF. Kanellopoulou et al. (2018) [21] and Luchowski et al. (2024) [24] examined the association between HbA1c and mortality, with the former focusing on stroke mortality in PAF patients under strict glycemic control, and the latter analyzing all-cause mortality in ischemic stroke patients. Similarly, Madhu et al. (2018) [27] and Kamel et al. (2022) [37] investigated long-term mortality risks, emphasizing the impact of glycemic control using insulin and intensive regimens, respectively. Functional recovery outcomes were the focus of Vakilian et al. (2018) [22], Nye (2022) [32], and Gofir et al. (2023) [25], highlighting the effectiveness of HbA1c monitoring and control strategies in post-stroke rehabilitation. The recurrence of stroke was addressed by Kezerle et al. (2020) [23] and Yang et al. (2016) [40], linking glycemic control and monitoring with reduced recurrence risks. Thromboembolic events, a significant complication, were explored by Wu et al. (2014) [26] and Haque (2019) [35], who reported the importance of continuous HbA1c monitoring in mitigating these risks. Studies such as those by Shafa et al. (2016) [30] and Nomani et al. (2016) [33] addressed stroke-related complications, noting that glycemic management played a crucial role in reducing post-stroke infections and other adverse outcomes. Finally, recovery outcomes were assessed by researchers like Hussein and Almansoob (2023) [43] and Sina et al. (2018) [36], who emphasized the value of diet, exercise, and glycemic monitoring in improving functional recovery and survival.

Table 3 summarizes the key findings of the studies included in the review, highlighting the relationship between HbA1c levels and outcomes in patients with ischemic stroke and AF. It details the impact of glycemic management strategies on mortality, stroke recurrence, functional recovery, and complications, emphasizing the importance of HbA1c as a prognostic marker and a target for intervention.

**Table 3:** Findings of studies included in the review

Author (year)	Key findings	Secondary outcome	Challenges
Glycemic control and mortality			
Kanellopoulou et al. (2018) [21]	Strict HbA1c control reduced mortality but increased the risk of hypoglycemia	Increased hospital admissions due to hypoglycemia	Managing hypoglycemia risk in the elderly population
Madhu et al. (2018) [27]	Better glycemic control led to lower mortality rates	Improved long-term cardiovascular outcomes	Adherence to treatment protocols and comorbidities
Nye (2022) [32]	Lower HbA1c levels were associated with reduced recurrence and improved recovery	Reduced stroke recurrence rates	Variability in management strategies across patients
Krezias (2015) [44]	Intensive glycemic control is associated with reduced mortality	Decreased incidence of diabetes-related complications	Adherence to strict glycemic control and risk of severe hypoglycemia
Afzal et al. (2021) [42]	Intensive glycemic control reduced complications and mortality rates	Reduced incidence of diabetes-related infections	Risk of severe hypoglycemia in elderly population
Kamel et al. (2022) [37]	Intensive glycemic control is associated with reduced mortality	Lowered incidence of post-stroke complications	Adherence to strict glycemic control and risk of severe hypoglycemia
Piñeiro et al. (2024) [38]	Tight glycemic control was linked to less severe stroke outcomes	Reduced stroke severity and improved outcomes	Difficulty in adjusting insulin dosages and managing comorbidities
Glycemic control and functional recovery			
Vakilian et al. (2018) [22]	Improved HbA1c levels were associated with better recovery outcomes	Enhanced rehabilitation success	Medication adherence issues in elderly patients such as cognitive decline or polypharmacy
Gofir et al. (2023) [25]	Patients with well-controlled HbA1c had better functional recovery	Improved mobility and daily activity scores	Risk of hypoglycemia in intensive glycemic control
Shafa et al. (2016) [30]	Better glycemic control reduced post-stroke complications	Shorter recovery times and fewer rehabilitation needs	Limited sample size and control over confounders
Shen et al. (2020) [45]	Tight glycemic control was linked to less severe stroke outcomes	Reduced neurological deficits	Difficulty in adjusting insulin dosages and managing comorbidities
Kezerle et al. (2022) [29]	Greater HbA1c variability is associated with worse recovery and higher mortality	Worse functional outcomes	Retrospective nature limited control over confounding factors
Hussein and Almansoob (2023) [43]	Better glycemic control and lifestyle intervention improved functional recovery	Enhanced quality of life and mobility	Lack of long-term follow-up and challenges with patient adherence to lifestyle changes
Hypoglycemia risk and challenges in management			
Luchowski et al. (2024) [24]	Strict HbA1c control reduced mortality but increased the risk of hypoglycemia	Increased frequency of severe hypoglycemic episodes	Managing hypoglycemia risk in the elderly population
Gofir et al. (2023) [25]	Medication adherence issues in elderly patients affected glycemic control	Difficulty maintaining glycemic stability in elderly patients	Cognitive decline or polypharmacy
Wu et al. (2014) [26]	Risk of hypoglycemia in intensive glycemic control	Increased hospitalizations due to hypoglycemia	Risk of hypoglycemia in intensive glycemic control
Kumar et al. (2022) [34]	HbA1c levels were a significant predictor of thromboembolic events	Risk of thromboembolic complications in patients with uncontrolled HbA1c	Difficulty in balancing blood glucose control with anticoagulation
Yenigalla et al. (2019) [46]	Tight glycemic control correlated with lower mortality rates	Risk of severe hypoglycemia in the elderly population	Risk of severe hypoglycemia in elderly population
Lei et al. (2015) [31]	Intensive glycemic control is associated with reduced mortality	Lower incidence of stroke-related complications	Increased risk of hypoglycemia with strict glycemic control
Lifestyle modifications			
Şenel and İnce (2021) [28]	Lifestyle modifications improved outcomes, but adherence was low	Long-term weight management and cardiovascular health	Low patient engagement in lifestyle changes
Sina et al. (2018) [36]	Better glycemic control led to improved functional recovery	Reduced post-stroke complications	Low adherence to lifestyle interventions
Stroke risk and recurrence			
Kezerle et al. (2020) [23]	Elevated HbA1c levels were predictive of stroke recurrence	Increased recurrence of strokes in high-HbA1c patients	High dropout rate from long-term follow-up
Yang et al. (2016) [40]	Greater HbA1c variability linked to increased stroke recurrence	Higher recurrence rates in patients with poor glycemic control	Retrospective nature limits causal inference and potential confounders
Haque (2019) [35]	HbA1c levels significantly correlated with thromboembolic risk	Increased risk of blood clot formation in uncontrolled patients	Lack of consistency in treatment regimens
Li et al. (2016) [41]	Metformin-based glycemic control decreased stroke-related complications	Reduced risk of post-stroke complications in diabetic patients	Low adherence to medication regimens and patient drop-out rates
Alfakeeh et al. (2024) [39]	Glycemic control reduced thromboembolic events after stroke	Decreased likelihood of developing blood clots	Limited follow-up duration and challenges in monitoring long-term effects

The studies reviewed exhibit varying levels of quality, ranging from low to high. The JBI Critical Appraisal Tools assign a score based on specific quality criteria for each study, with each criterion scored as "yes," "no," or "unclear." The final score is calculated as the percentage of "yes" responses over the total possible points. High-quality studies have scores above 70%, indicating rigorous methodology and strict adherence to appraisal criteria. Medium-quality studies score between 50% and 70%, with minor issues, such as unclear participant selection or limitations in statistical analysis. Low-quality studies have scores below 50%, often with methodological issues or significant biases affecting the reliability of findings. These studies may have issues with study design, participant selection, confounding factors, or statistical analysis.

The critical appraisal of the studies reveals varying quality levels. Wu et al. (2014) [26] is of high quality (71.42%) with clear inclusion criteria and reliable outcome measures, but it lacks strategies for confounding. Shafa et al. (2016) [30] and Lei et al. (2015) [31] are of medium quality (57.14%) due to unclear inclusion criteria and failure to address confounding. Lei et al. (2015) [31] is of low quality (42.85%) due to unreliable exposure measurement and inadequate confounding control. Despite some strengths, key limitations in handling confounders and clarity in sample selection affect the overall validity of these studies.

The studies reviewed demonstrate strong methodological rigor, with most scoring high quality (63.60% and above). Kanellopoulou et al. (2018) [21], Kezerle et al. (2020) [23], and Luchowski et al. (2024) [24] all identified confounding factors and utilized valid and reliable exposure measures, though follow-up was incomplete in some cases. Madhu et al. (2018) [27] and Nye (2022) [32] showed challenges with follow-up, which impacted their total scores. Studies like Yenigalla et al. (2019) [46] and Sina et al. (2018) [36] reported sufficient outcome measurement but lacked adequate follow-up time. Kumar et al. (2022) [34] and Kamel et al. (2022) [37] had robust methodologies with high follow-up completion rates. Overall, while these studies have methodological strengths, issues such as follow-up time and incomplete follow-up in some studies limit their conclusions.

Vakilian et al. (2018) [22] exhibited high quality with low risks of bias across all categories, ensuring reliable results. Gofir et al. (2023) [25] showed moderate quality, with high selection and reporting biases, but low performance and detection biases. Şenel and İnce (2021) [28] demonstrated moderate quality due to high risks of detection and attrition bias, as well as concerns about selective reporting. Nomani et al. (2016) [33] also had moderate quality, with low selection, performance, and detection bias, but concerns about attrition and selective reporting. Li et al. (2016) [41] displayed moderate quality due to low biases in most areas, but high attrition bias significantly affected the study's reliability. Overall, studies with low biases in key areas (e.g., Vakilian et al. (2018)) were of higher quality, while those with high or some concerns (e.g., Gofir et al. (2023), Şenel and İnce (2021), and Li et al. (2016)) had moderate quality.

All-Cause Mortality

Glycemic control has been found to have a limited impact on elderly patients with multiple comorbidities [48]. For instance, Kezerle et al. (2020) [23] found that strict glycemic control improved recovery outcomes in elderly patients with ischemic stroke and diabetes, but was less pronounced in those with hypertension and heart disease. Haque (2019) [35] found that intensive glycemic control had a diminished effect in patients with multiple comorbidities, such as kidney disease or obesity. These patients struggled to maintain blood sugar levels within the recommended range due to factors like polypharmacy, limited mobility, and renal dysfunction. Patients with kidney disease had a 30% increased risk of severe hypoglycemia despite intensive glycemic control, and only a 10% reduction in stroke-related complications compared to 25% in patients without kidney issues [45]. Social determinants of health (SDOH) can significantly influence the outcomes of glycemic control in elderly populations, with studies showing that individuals from lower socioeconomic backgrounds are less likely to adhere to glycemic control regimens [39]. A comprehensive approach that includes health education, access to care, and support systems could improve outcomes, especially in populations with greater vulnerability.

Li et al. (2016) [41] found that individuals with ischemic stroke and PAF are more likely to die from all causes due to high HbA1c values, indicating poor glycemic management. This aligns with previous research showing a correlation between poor diabetes management and increased death rates in people with heart and brain disorders. However, age, comorbidities, and cardiovascular disease severity may impact the effect of glycemic management on mortality.

Stroke-Related Mortality

Diabetic patients, particularly those with arrhythmias, experience complex vascular damage due to chronic hyperglycemia, oxidative stress, inflammation, advanced glycation end products (AGEs), hypercoagulability, and atherosclerosis. Diabetes accelerates atherosclerosis by promoting lipid accumulation, inflammatory responses, and endothelial damage [49]. Stroke patients with arrhythmias, such as PAF, experience more complex vascular damage mechanisms, including thromboembolism, increased systemic inflammation, and hemodynamic changes [45]. Glycemic control is crucial for reducing endothelial damage, preventing recurrent stroke, monitoring hypoglycemia risk, and setting individualized targets [29]. For patients with ischemic stroke and concurrent arrhythmias like AF, glycemic control strategies become more nuanced, considering hypoglycemia risk, anticoagulation considerations, and individualized targets [43]. Understanding the pathophysiological mechanisms of vascular damage in diabetic stroke patients highlights the complexity of managing these patients. Tight glycemic control can prevent further damage and reduce stroke risk, but a more tailored approach is necessary for patients with arrhythmias like AF [22]. Interventions should aim to prevent vascular damage and optimize anticoagulation therapy for optimal clinical outcomes.

Functional Recovery

The timing and intensity of rehabilitation are crucial for the recovery of ischemic stroke patients, influenced by factors such as age, stroke severity, and glycemic control [50]. Early rehabilitation can improve functional independence and reduce long-term disability, especially in younger patients or those with less severe strokes [21]. However, elderly patients or those with severe strokes may experience delayed recovery due to medical complications [27]. The intensity of rehabilitation, such as more frequent physical therapy sessions, is associated with better functional recovery. However, in the context of diabetes and glycemic control, overexertion can lead to hypoglycemia or exacerbate cardiovascular complications [44]. Older patients face more challenges in stroke recovery due to physiological decline, diminished neuronal plasticity, slower healing processes, and multiple comorbidities. Poor glycemic control in elderly stroke patients has been associated with delayed recovery, increased risk of complications, and worse outcomes, including higher mortality. Tight glycemic control in severe stroke patients may improve recovery outcomes [37].

Stroke Recurrence

Poor glycemic control, particularly in diabetes patients, can significantly affect blood viscosity and vascular function, increasing the risk of adverse cardiovascular outcomes like stroke, myocardial infarction, and other ischemic events [46]. Chronic hyperglycemia also directly affects vascular health, contributing to atherosclerosis and increasing the risk of vascular occlusion, which can lead to stroke or other ischemic conditions [31]. The role of glycemic control versus cardiovascular risk management is crucial in preventing cardiovascular events. While strict glycemic control is important for preventing long-term complications of diabetes, overly tight glycemic control can increase the risk of hypoglycemia, which has adverse cardiovascular effects [28]. The benefit of glycemic control lies in reducing microvascular damage, maintaining proper blood viscosity, and preventing atherosclerosis. However, controlling blood glucose alone may not be sufficient for optimal cardiovascular health, especially in patients with other risk factors [36]. Cardiovascular risk management, including blood pressure and cholesterol management, is essential for reducing broader vascular risk. A multidisciplinary approach is essential for improving outcomes and reducing the burden of cardiovascular events in diabetic patients [51].

Thromboembolic Events

Chronic uncontrolled diabetes can lead to vascular damage and an increased risk of thrombotic events due to reduced nitric oxide, increased adhesion molecules, and overproduction of reactive oxygen species [52]. This inflammation promotes plaque formation, rupture, and clot formation. Chronic hyperglycemia increases vascular permeability, allowing pro-inflammatory cytokines to leak into tissues [53]. The interplay between glycemic control and antithrombotic therapy is complex and crucial in managing stroke patients, especially those with diabetes [42]. Tight glycemic control improves stroke outcomes, reduces recurrence, and enhances recovery, but it increases the risk of hypoglycemia, particularly when combined with anticoagulants like warfarin or direct oral anticoagulants. Poor glycemic control can exacerbate thromboembolic risks, making it difficult to balance with anticoagulation therapy [37]. Studies show that intensive glycemic control can lead to better functional recovery, but it requires careful monitoring to avoid bleeding complications [38]. Medication adherence is a challenge, particularly in elderly or cognitively impaired patients, complicating the simultaneous management of both therapies [25]. Moreover, interactions between antithrombotic medications and glycemic agents can affect both efficacy and safety [30]. Close monitoring of both blood glucose and antithrombotic levels is essential to optimize patient outcomes. Hypoglycemia and medication non-adherence further complicate treatment [54]. Future strategies should focus on balancing these therapies to prevent adverse events while ensuring long-term health benefits.

Cardiovascular Mortality

Glycemic control, particularly through HbA1c management, is essential for improving vascular health and reducing cardiovascular mortality, especially in patients with diabetes or stroke [55]. Studies such as Kanellopoulou et al. (2018) [21] and Yenigalla et al. (2019) [46] show that tight glycemic control reduces mortality and improves recovery outcomes. Poor glycemic control, as seen in Kezerle et al. (2020) [23], is linked to increased stroke recurrence, emphasizing its role in vascular health. Afzal et al. (2021) [42] found that intensive glycemic control reduces mortality and stroke complications. However, managing other cardiovascular risk factors, like blood pressure and lipid levels, is also crucial. Blood pressure control significantly reduces stroke and heart disease risks, while lipid management, particularly lowering low-density lipoprotein (LDL) cholesterol, decreases cardiovascular disease incidence [37]. Piñeiro et al. (2024) [38] highlight the importance of blood pressure and lipid management alongside glycemic control. Combining these strategies leads to better overall cardiovascular outcomes. Tight management of all three - glycemic control, blood pressure, and lipids - is essential for comprehensive cardiovascular risk reduction.

Paroxysmal Atrial Fibrillation

AGEs play a significant role in atrial remodeling and cardiovascular complications, particularly in patients with diabetes. AGEs are formed when glucose binds to proteins, lipids, or nucleic acids, leading to cross-linking and structural changes that promote inflammation, fibrosis, and endothelial dysfunction [56]. In the context of atrial remodeling, AGEs contribute to electrical conduction abnormalities and arrhythmias by stiffening the atrial myocardium and increasing fibrosis. Studies such as Wu et al. (2014) [26] highlight the role of glycemic control in improving recovery outcomes, suggesting that controlling HbA1c can reduce AGE-related damage. Furthermore, diabetes accelerates atherosclerosis and plaque formation by increasing AGE deposition in the vascular walls, leading to endothelial dysfunction and increased inflammation [31]. This promotes the development of plaques that narrow blood vessels and enhance the risk of thromboembolic events, as noted in studies like Sina et al. (2018) [36], which link poor glycemic control to worsened vascular health. AGEs also impair vascular smooth muscle function, contributing to arterial stiffness and promoting atherosclerotic plaque instability. Thus, diabetes-induced AGE formation accelerates atherosclerosis, making effective glycemic control essential for mitigating cardiovascular risks, as seen in studies like Alfakeeh et al. (2024) [39].

Overall Impact of HbA1c Control on Patient Outcomes

Holistic management of diabetes and cardiovascular disease emphasizes controlling HbA1c, blood pressure, and lipid levels [57]. Piñeiro et al. (2024) [38] highlight that tight glycemic control improves recovery outcomes and reduces mortality, but achieving HbA1c targets can be challenging due to the risk of hypoglycemia, particularly in elderly patients on multiple medications. Nye (2022) [32] stresses that medication adherence and lifestyle changes are significant hurdles in managing both diabetes and cardiovascular risk. Blood pressure control and lipid management are equally important, with guidelines recommending blood pressure below 140/90 mmHg and statin therapy to reduce cardiovascular events [25]. Kezerle et al. (2022) [29] show that glycemic control can mitigate atherosclerotic risk and stroke recurrence, but careful management is required to balance treatment goals. Personalized care, frequent monitoring, and patient education are essential for optimal outcomes in this high-risk population.

Table 4 provides an overview of studies excluded from the systematic review, along with the specific reasons for exclusion. Exclusions were based on predefined criteria, such as non-English language, irrelevance to the research question, insufficient data on HbA1c outcomes, or study designs that did not meet inclusion requirements (e.g., case reports or studies with incomplete methodologies). The excluded studies highlight the rigorous selection process undertaken to ensure the reliability and relevance of the review findings.

**Table 4:** Excluded studies

Study	Study title	Reason for exclusion	Explanation
Bateman et al. (2016) [58]	Effect of glucagon-like peptide-1 (GLP-1) on glycemic control and left ventricular function in patients undergoing coronary artery bypass grafting.	Irrelevant outcome	Focused on GLP-1 effects on glycemic control and heart function in a coronary artery bypass grafting population, which does not relate to stroke recovery or atrial fibrillation.
Huijben et al. (2018) [59]	Vascularization pattern after ischemic stroke is different in control versus diabetic rats: relevance to stroke recovery.	Animal model	This study used animal models (rats) and focused on vascularization patterns post-stroke, which does not provide relevant data for human clinical outcomes or glycemic control after stroke.
Lattanzi et al. (2016) [60]	Comparative analysis of the neurovascular injury and functional outcomes in experimental stroke models in diabetic Goto-Kakizaki rats.	Animal model	This study focused on diabetic rats in experimental stroke models and did not investigate human stroke outcomes or glycemic control in humans.
Li et al. (2013) [61]	Beneficial effects of acute inhibition of the oxidative pentose phosphate pathway in the failing heart.	Irrelevant outcome	The study focused on heart failure mechanisms rather than stroke, atrial fibrillation, or stroke recovery.
Prakash et al. (2013) [62]	Glycosylated hemoglobin and functional outcome after acute ischemic stroke.	Outdated	This study was published in 2013 and does not include newer treatment protocols or modern data on stroke recovery or glycemic control.
Sokos et al. (2007) [63]	Role of hypoxia-inducible factor 1 in hyperglycemia-exacerbated blood-brain barrier disruption in ischemic stroke.	Irrelevant focus	Although relevant to stroke, it focuses on blood-brain barrier disruption in hyperglycemia, not directly on glycemic control or functional outcomes in stroke recovery.
Vimercati et al. (2014) [64]	36th International Symposium on Intensive Care and Emergency Medicine: Brussels, Belgium. 15-18 March 2016.	Conference abstract	This is an abstract from a conference, not a full peer-reviewed study. Therefore, it lacks the depth and detail required for inclusion in a systematic review.
Wang et al. (2019) [65]	Effect of sitagliptin on the echocardiographic parameters of left ventricular diastolic function in patients with type 2 diabetes: a subgroup analysis of the PROLOGUE study.	Irrelevant outcome	Focused on cardiovascular function and diabetes, but not related to stroke recovery, atrial fibrillation, or functional outcomes post-stroke.
Yamada et al. (2017) [66]	Variation in general supportive and preventive intensive care management of traumatic brain injury: a survey in 66 neurotrauma centers participating in the Collaborative European NeuroTrauma Effectiveness Research in Traumatic Brain Injury (CENTER-TBI) study.	Irrelevant focus	The study addresses traumatic brain injury (TBI) management, which is distinct from ischemic stroke or atrial fibrillation, thus not relevant to the review's scope.
Barra et al. (2024) [67]	Lower serum osteocalcin concentrations in patients with type 2 diabetes and relationships with vascular risk factors among patients with coronary artery disease.	Irrelevant focus	This study is focused on osteocalcin levels, vascular risk factors, and coronary artery disease in type 2 diabetes, without addressing stroke, atrial fibrillation, or glycemic control after stroke.
Darwish et al. (2019) [68]	White matter hyperintensity volume in pre-diabetes, diabetes, and normoglycemia.	Irrelevant outcome	Focuses on the white matter changes in pre-diabetes and diabetes, which does not address stroke recovery or functional outcomes related to glycemic control in ischemic stroke.
Grosu et al. (2021) [69]	HbA1c level is associated with the development of heart failure with recovered ejection fraction in hospitalized heart failure patients with type 2 diabetes.	Irrelevant focus	This study addresses heart failure in diabetes and HbA1c levels but does not provide data on stroke outcomes or glycemic control in stroke patients.
Yang et al. (2023) [70]	Medication profiles at hospital discharge predict poor outcomes after acute ischemic stroke.	Irrelevant outcome	The study is valuable, but it overlaps with more current data focusing specifically on medication profiles and stroke outcomes rather than glycemic control.
Yang et al. (2023) [71]	Long-term glycemic variability predicts compromised development of heart failure with improved ejection fraction: a cohort study.	Irrelevant outcome	Focuses on heart failure outcomes in patients with type 2 diabetes and does not address stroke recovery or functional outcomes after ischemic stroke.

The JBI Critical Appraisal Checklist for cross-sectional studies was employed to evaluate the methodological quality and rigor of the included studies (Table 5). This tool assesses various aspects, such as clarity in research objectives, appropriateness of study design, sampling strategies, data collection methods, and reliability of outcome measurements.

**Table 5:** Joanna Briggs Institute (JBI) Critical Appraisal Checklist for cross-sectional studies

Studies	Were the criteria for inclusion in the sample clearly defined?	Were the study subjects and the setting described in detail?	Was the exposure measured in a valid and reliable way?	Were objective, standard criteria used for measurement of the condition?	Were confounding factors identified?	Were strategies to deal with confounding factors stated?	Were the outcomes measured in a valid and reliable way?	Was appropriate statistical analysis used?	Total quality assessment score	Total quality
Wu et al. (2014) [26]	Yes	Yes	Yes	No	Yes	No	Yes	Yes	71.42%	High quality
Shafa et al. (2019) [30]	No	Yes	Yes	No	Yes	No	Yes	Yes	57.14%	Medium quality
Lei et al. (2015) [31]	Yes	No	No	Yes	No	No	Yes	Yes	42.85%	Low quality
Hussein and Almansoob (2023) [43]	No	Yes	Yes	No	Yes	No	Yes	Yes	57.14%	Medium quality

The critical appraisal of the studies reveals varying quality levels. Wu et al. (2014) [26], rated as high quality (71.42%), demonstrated strengths such as clear inclusion criteria and reliable outcome measures. However, the lack of strategies for controlling confounders limited its internal validity. Shafa et al. (2016) [30] and Lei et al. (2015) [31], both rated as medium quality (57.14%), exhibited common weaknesses, including unclear inclusion criteria and insufficient attention to confounding, which weakened the reliability of their findings. Lei et al. (2015) [31] was further rated as low quality (42.85%) due to unreliable exposure measurement and inadequate control of confounders, further undermining its validity. The quality assessments had significant implications for the overall review. Medium- and low-quality studies were treated with caution in the synthesis of findings, with their results given less weight due to the limitations in their methodologies. These studies were still included in the review to provide a broader perspective, but their conclusions were interpreted more conservatively. No high-quality studies directly contradicted the findings of lower-quality studies. However, the methodological flaws in the medium- and low-quality studies suggest that their results may not be as reliable, and further research with stronger controls will be needed to validate their findings.

Table 6 provides the JBI Critical Appraisal Checklist for cohort studies. This checklist assesses the methodological quality of cohort studies to determine the validity and reliability of their findings. It includes key criteria, such as participant selection, exposure measurement, confounding factor adjustment, outcome assessment, and follow-up adequacy.

**Table 6:** Joanna Briggs Institute (JBI) Critical Appraisal Checklist for cohort studies

Studies	Were the two groups similar and recruited from the same population?	Were the exposures measured similarly to assign people to both exposed and unexposed groups?	Was the exposure measured in a valid and reliable way?	Were confounding factors identified?	Were strategies to deal with confounding factors stated?	Were the groups/participants free of the outcome at the start of the study?	Were the outcomes measured in a valid and reliable way?	Was the follow-up time reported and sufficient to be long enough for outcomes to occur?	Was the follow-up complete, and if not, were the reasons for loss to follow-up described and explored?	Were strategies to address incomplete follow-up utilized?	Was appropriate statistical analysis used?	Total quality assessment score	Total quality
Kanellopoulou et al. (2018) [21]	Yes	Yes	Yes	Yes	Yes	Yes	Yes	No	No	No	Yes	72.70%	High quality
Kezerle et al. (2020) [23]	Yes	Yes	Yes	No	Yes	Yes	Yes	Yes	No	No	Yes	81.80%	High quality
Luchowski et al. (2024) [24]	Yes	Yes	Yes	No	Yes	Yes	Yes	No	Yes	No	Yes	81.80%	High quality
Madhu et al. (2018) [27]	Yes	Yes	Yes	Yes	Yes	Yes	Yes	No	No	No	Yes	63.60%	High quality
Kezerle et al. (2022) [29]	Yes	Yes	Yes	No	Yes	Yes	Yes	Yes	No	No	Yes	72.70%	High quality
Yenigalla et al. (2019) [46]	Yes	Yes	Yes	No	Yes	Yes	Yes	No	Yes	No	Yes	72.70%	High quality
Nye (2022) [32]	Yes	Yes	Yes	Yes	Yes	Yes	Yes	No	No	No	Yes	63.60%	High quality
Kumar et al. (2022) [34]	Yes	Yes	Yes	No	Yes	Yes	Yes	No	Yes	No	Yes	81.80%	High quality
Haque (2019) [35]	Yes	Yes	Yes	Yes	Yes	Yes	Yes	No	No	No	Yes	63.60%	High quality
Sina et al. (2018) [36]	Yes	Yes	Yes	No	Yes	Yes	Yes	Yes	No	No	Yes	72.70%	High quality
Kamel et al. (2022) [37]	Yes	Yes	Yes	No	Yes	Yes	Yes	No	Yes	No	Yes	81.80%	High quality
Piñeiro et al. (2024) [38]	Yes	Yes	Yes	No	Yes	Yes	Yes	No	Yes	No	Yes	81.80%	High quality
Alfakeeh et al. (2024) [39]	Yes	Yes	Yes	Yes	Yes	Yes	Yes	No	No	No	Yes	63.60%	High quality
Yang et al. (2016) [40]	Yes	Yes	Yes	No	Yes	Yes	Yes	No	Yes	No	Yes	81.80%	High quality
Afzal et al. (2021) [42]	Yes	Yes	Yes	Yes	Yes	Yes	Yes	No	No	No	Yes	63.60%	High quality
Krezias (2015) [44]	Yes	Yes	Yes	No	Yes	Yes	Yes	Yes	No	No	Yes	72.70%	High quality
Shen et al. (2020) [45]	Yes	Yes	Yes	No	Yes	Yes	Yes	No	Yes	No	Yes	81.80%	High quality

The studies reviewed demonstrate strong methodological rigor, with most scoring high quality (63.60% and above). Despite some issues, the studies reviewed demonstrate overall methodological rigor, with high-quality ratings. Studies like Kanellopoulou et al. (2018) [21], Kezerle et al. (2020) [23], and Luchowski et al. (2024) [24] identified confounding factors and used valid exposure measures; yet, some faced incomplete follow-up. This limits the conclusions drawn but does not detract from their high-quality ratings, as the exposure measurement and confounder identification were robust. On the other hand, Madhu et al. (2018) [27] and Nye (2022) [32] had follow-up challenges, which lowered their scores due to the critical role of follow-up in cohort studies. Yenigalla et al. (2019) [46] and Sina et al. (2018) [36] had sufficient outcome measurement but lacked adequate follow-up time, which affects long-term generalizability. Kumar et al. (2022) [34] and Kamel et al. (2022) [37] had high follow-up completion rates, justifying their higher scores. Although all studies scored well on statistical analysis, weaknesses in follow-up and confounder management could undermine the validity of the statistical results. Missing data from incomplete follow-up can introduce bias, and failure to control for confounders could affect the internal validity of the findings. These gaps in methodology are critical to note when assessing overall study quality, as they influence the reliability and generalizability of the conclusions.

Table 7 summarizes the assessment of the risk of bias for included RCTs using the Cochrane Risk of Bias checklist.

**Table 7:** Cochrane Risk of Bias Checklist for randomized controlled trials

Studies	Allocation concealment (selection bias)	Binding of participation and personnel (performance bias)	Binding of outcome assessment (detection bias)	Incomplete outcome data (attrition bias)	Selective reporting (reporting bias)	Overall quality
Vakilian et al. (2018) [22]	Low	Low	Low	Low	Low	High
Gofir et al. (2023) [25]	High	Low	Low	Low	High	Low
Şenel and İnce (2021) [28]	Low	Low	High	High	Some concern	Low
Nomani et al. (2016) [33]	Low	Low	Low	Some concern	Some concern	Some concern
Li et al. (2016) [41]	Low	Low	Low	High	Low	High

Vakilian et al. (2018) [22] exhibited high quality, with low risks of bias across all categories, ensuring reliable results. Gofir et al. (2023) [25] showed moderate quality, with high selection and reporting biases, but low performance and detection biases. Şenel and İnce (2021) [28] demonstrated moderate quality due to high risks of detection and attrition bias, as well as concerns about selective reporting. Nomani et al. (2016) [33] also had moderate quality, with low selection, performance, and detection bias, but concerns about attrition and selective reporting. Li et al. (2016) [41] displayed moderate quality due to low biases in most areas, but high attrition bias significantly affected the study’s reliability. Overall, studies with low biases in key areas (Vakilian et al. (2018)) were of higher quality, while those with high or some concerns (Gofir et al. (2023), Şenel and İnce (2021), and Li et al. (2016)) had moderate quality.

Discussion

Glycemic Control and Mortality

Elevated HbA1c levels significantly increase the risk of all-cause mortality in patients with paroxysmal AF and ischemic stroke due to poor glycemic management. Sina et al. (2018) [36] support this, linking poor outcomes and higher mortality to elevated HbA1c. Similarly, Kumar et al. (2022) [34] highlight that poor diabetes control exacerbates cardiovascular mortality by accelerating atherosclerosis and vascular stiffness. Wu et al. (2014) [26] contrast this, suggesting that, in paroxysmal AF patients, anticoagulation therapy may mitigate the mortality impact of hyperglycemia despite its contribution to stroke recurrence. Additionally, Haque (2019) [35] demonstrates that PAF, combined with poor glycemic management, substantially increases cardiovascular mortality.

Stroke Recurrence

High HbA1c levels are a known risk factor for stroke recurrence, as reported by Wu et al. (2014) [26] and Alfakeeh et al. (2024) [39]. The link between elevated HbA1c and thromboembolic risks, including stroke recurrence, underscores the need for comprehensive management. Kezerle et al. (2020) [23] emphasize integrating glycemic control with antithrombotic therapy for AF patients. Supporting this, Sina et al. (2018) [36] found that glycemic control, combined with blood pressure and cholesterol management, plays a vital role in reducing recurrence risk. Şenel and İnce (2021) [28] highlight the endothelial dysfunction caused by elevated HbA1c, which increases thromboembolic risks.

Functional Recovery

Lower HbA1c levels are associated with improved rehabilitation outcomes after ischemic stroke, as shown by Shen et al. (2020) [45]. Kezerle et al. (2022) [29] highlight that addressing other cardiovascular risks, such as smoking cessation and lipid control, alongside glycemic management, is crucial for functional recovery. Luchowski et al. (2024) [24] emphasize that age, stroke severity, and rehabilitation timing significantly influence outcomes, advocating for a multifactorial approach. Piñeiro et al. (2024) [38] also suggest that comprehensive glycemic management improves recovery by reducing complications related to hyperglycemia.

The contrasting findings between Sina et al. (2018) [36] and Xu et al. (2012) [72] may arise from differences in study populations, methodologies, and confounding factors. Sina et al. (2018) [36] focused broadly on ischemic stroke patients, emphasizing that elevated HbA1c levels worsen outcomes and increase mortality. In contrast, Xu et al. (2012) [72] highlighted stroke recurrence risks while suggesting that anticoagulation therapy in AF patients could mitigate hyperglycemia's impact on mortality. Variations in baseline characteristics, such as the prevalence of AF, use of anticoagulation, and other cardiovascular therapies, may account for these differences. Furthermore, methodological aspects, including differences in follow-up duration, statistical adjustments for comorbidities, and glycemic control measurements, could also explain the discrepancies. A critical exploration of these factors could clarify these findings and inform targeted interventions.

A multifactorial approach and comprehensive strategy for managing ischemic stroke and AF involves several key components. Targeted antithrombotic therapy is crucial for reducing thromboembolic risks, with individualized anticoagulation regimens tailored to patient-specific risk profiles using the CHA_2_DS_2_-VASc and HAS-BLED scoring systems [73]. Equally important is optimized glycemic management, which includes maintaining HbA1c within an optimal range through personalized care plans involving CGM, individualized medication regimens, and lifestyle modifications [74]. Additionally, cardiovascular risk factor control, such as managing hypertension, dyslipidemia, and promoting smoking cessation, can be achieved through statins, antihypertensives, and behavioral counseling [75].

Stroke rehabilitation plays a significant role in improving functional outcomes, with programs designed to consider glycemic control, age, stroke severity, and early intervention timelines [76]. Addressing SDOH is essential, as socioeconomic and psychosocial barriers to care can impact outcomes; strategies include enhancing healthcare access, providing education, and supporting adherence to prescribed interventions [77]. An integrated multidisciplinary care model further strengthens this approach by coordinating efforts among cardiologists, endocrinologists, neurologists, and rehabilitation specialists [78]. Lastly, patient-centric education and support empower individuals to self-manage their condition effectively through tailored educational programs, psychological support, and regular follow-ups. By integrating these components, a comprehensive strategy can improve mortality rates, reduce stroke recurrence, and enhance recovery outcomes for patients [79].

Medication adherence and lifestyle changes are crucial confounding variables that significantly influence glycemic control and stroke outcomes. Poor adherence to prescribed medications, such as antidiabetics and anticoagulants, can lead to uncontrolled blood sugar levels and increased stroke recurrence, as seen in studies by Haque (2019) [35]. Lifestyle factors, including diet, physical activity, and smoking, also impact outcomes. Nomani et al. (2016) [33] highlighted that poor dietary habits and lack of physical activity contribute to elevated HbA1c levels, which worsen stroke outcomes. Interventions to improve adherence, such as patient education and simplified medication regimens, have been shown to enhance glycemic control and reduce mortality [37]. Furthermore, lifestyle modifications, such as increased physical activity and smoking cessation, play a critical role in mitigating cardiovascular risks [38]. To reduce biases in future research, it is essential to account for these confounders and explore strategies to improve adherence and lifestyle changes through integrated care approaches.

Future studies should consider utilizing CGM real-time data on glycemic fluctuations, improving accuracy over HbA1c. Additionally, a mixed-methods approach, combining quantitative and qualitative data, could identify barriers to lifestyle changes and adherence. Incorporating biomarker analysis could also shed light on the mechanisms linking glycemic control to stroke recurrence and mortality.

The observational nature of many studies limits the ability to establish causality, making it difficult to definitively link glycemic control to stroke outcomes. This introduces confounding bias, where unaccounted-for factors (e.g., medication adherence or lifestyle changes) could influence both variables. To mitigate this, RCTs should be employed, as they are better suited for establishing cause-and-effect relationships. Furthermore, the lack of control over confounders in observational studies could skew results, as factors like physical activity, diet, and socioeconomic status may vary widely. Future research should adopt prospective cohort studies that rigorously track these confounders through electronic health records, wearables, or surveys. The reliance on HbA1c alone to measure glycemic control is another limitation, as it may not reflect day-to-day glucose fluctuations. Using CGM could provide more precise, real-time data on blood sugar fluctuations and their impact on stroke outcomes. By addressing these limitations through advanced study designs and measurement methods, future research can reduce bias and offer more reliable insights into the relationship between glycemic control and stroke outcomes.

Recent advancements in AF management and glycemic control offer valuable insights into improving patient outcomes, complementing the studies referenced earlier.

In AF management, a 2023 guideline update from the American College of Cardiology (ACC) emphasizes the importance of integrated care, including lifestyle modifications (weight loss, physical activity), and personalized anticoagulation therapy [80]. Innovations in stroke prevention strategies, including better anticoagulation control through NOACs (non-vitamin K antagonist oral anticoagulants), have led to improved outcomes in patients with AF.

For glycemic control, recent innovations post-2022 highlight the role of CGM, which provides real-time feedback on blood glucose fluctuations, allowing for more precise diabetes management. Hybrid closed-loop systems, combining CGM with insulin pumps, have been shown to improve time in range (TIR), which is crucial for reducing long-term complications.

These innovations offer promising improvements in stroke risk management and diabetes control, suggesting that integrating newer technologies into clinical practice may reduce stroke recurrence and mortality in patients with AF and ischemic stroke.

High HbA1c levels can increase mortality and stroke recurrence by promoting endothelial dysfunction, atherosclerosis, and thromboembolic events. Elevated blood sugar also induces inflammation and oxidative stress, which impair vascular health and hinder recovery. In patients with AF, poor glycemic control raises the risk of clot formation, worsening outcomes. Additionally, hyperglycemia disrupts blood flow and oxygen delivery to tissues, impairing stroke recovery. Overall, these factors contribute to poorer functional recovery and higher risks of adverse events.

## Conclusions

The observational nature of this study limits its ability to establish causal relationships between high HbA1c levels and outcomes like mortality, stroke recurrence, and functional recovery. Therefore, further research is necessary to confirm these findings and explore potential causal links. Future studies should focus on the impact of dynamic glycemic control, assessing how real-time adjustments may affect long-term outcomes in patients. Additionally, investigating the effects of targeted interventions for patients with dual diagnoses of ischemic stroke and PAF could provide valuable insights into improving patient management and recovery. Higher HbA1c levels worsen functional recovery outcomes, necessitating a multidisciplinary strategy involving diabetes educators, cardiologists, stroke rehabilitation specialists, and others to optimize glycemic control and rehabilitation.
